# Therapeutic Rescue of Misfolded Mutants: Validation of Primary High Throughput Screens for Identification of Pharmacoperone Drugs

**DOI:** 10.1371/journal.pone.0022784

**Published:** 2011-07-27

**Authors:** Jo Ann Janovick, Byung S. Park, P. Michael Conn

**Affiliations:** 1 Divisions of Reproductive Sciences and Neuroscience, Oregon National Primate Research Center, Beaverton, Oregon, United States of America; 2 Division of Biostatistics, Department of Public Health and Preventive Medicine, Oregon Health and Science University, Portland, Oregon, United States of America; 3 Departments of Physiology and Pharmacology, Cell Biology and Human Development and Obstetrics and Gynecology, Oregon Health & Science University, Portland, Oregon, United States of America; Aston University, United Kingdom

## Abstract

**Background:**

Functional rescue of misfolded mutant receptors by small non-peptide molecules has been demonstrated. These small, target-specific molecules (pharmacological chaperones or “pharmacoperones”) serve as molecular templates, promote correct folding and allow otherwise misfolded mutants to pass the scrutiny of the cellular quality control system (QCS) and be expressed at the plasma membrane (PM) where they function similarly to wild type (WT) proteins. In the case of the gonadotropin releasing hormone receptor (GnRHR), drugs that rescue one mutant typically rescue many mutants, even if the mutations are located at distant sites (extracellular loops, intracellular loops, transmembrane helices). This increases the value of these drugs. These drugs are typically identified, post hoc, from “hits” in screens designed to detect antagonists or agonists. The therapeutic utility of pharmacoperones has been limited due to the absence of screens that enable identification of pharmacoperones *per se*.

**Methods and Findings:**

We describe a generalizable primary screening approach for pharmacoperone drugs based on measurement of gain of activity in stable HeLa cells stably expressing the mutants of two different model G-protein coupled receptors (GPCRs) (hGnRHR[E^90^K] or hV2R[L^83^Q]). These cells turn off expression of the receptor mutant gene of interest in the presence of tetracycline and its analogs, which provides a convenient means to identify false positives.

**Conclusions:**

The methods described and characterized here provide the basis of novel primary screens for pharmacoperones that detect drugs that rescue GPCR mutants of specific receptors. This approach will identify structures that would have been missed in screens that were designed to select only agonists or antagonists. Non-antagonistic pharmacoperones have a therapeutic advantage since they will not compete for endogenous agonists and may not have to be washed out once rescue has occurred and before activation by endogenous or exogenous agonists.

## Introduction

G protein-coupled receptors (GPCRs), which include the gonadotropin releasing hormone (GnRH) receptor (GnRHR) and vasopressin type 2 receptor (V2R), comprise the largest family of validated drug targets; 30–50% of approved drugs derive their benefits by selective targeting of GPCRs [1]. Mutations in GPCRs are known to be responsible for over 30 disorders, including cancers, heritable obesity and endocrine diseases. Normally, GPCRs are subjected to a stringent quality control system (QCS) in the endoplasmic reticulum (ER). The QCS insures that only correctly folded proteins enter the pathway leading to the plasma membrane (PM). This system consists of both protein chaperones that retain misfolded proteins and enzyme-like proteins that participate in catalysis of the folding process. It has become apparent that point mutations may result in the production of misfolded and disease-causing proteins that are unable to reach their functional destinations in the cell because they are retained by the QCS even though they may retain function.

Pharmacoperone drugs (from “pharmacological chaperone”) are small molecules that enter cells and serve as molecular scaffolding in order to cause otherwise-misfolded mutant proteins to fold and route correctly within the cell. Many pharmacoperones are also agonists or antagonists because they have come from high throughput screens that were originally designed with a view toward identification of such congeners as lead drug candidates, not pharmacoperones as such. Pharmacoperone activity has been identified in these targets *post hoc* by us [2], [3] and others [4] for the GnRHR and V2R systems, respectively. Valuable drugs which effect the trafficking of GPCRs may have been overlooked because of this limitation.

In principle, the pharmacoperone-rescue approach applies to a diverse array of human diseases that result from protein misfolding – among these are cystic fibrosis [5], [6], [7], [8] hypogonadotropic hypogonadism (HH, [9]), nephrogenic diabetes insipidus [10], [11], [12], retinitis pigmentosa [13], hypercholesterolemia, cataracts [14], neurodegenerative diseases (Huntington's, Alzheimer's, Parkinson's [15], [16], [17], [18], [19]) and particular cancers [20]. In the case of certain proteins (e.g. the GnRHR, V2R and rhodopsin), this approach has succeeded with a striking number of different mutants [21], supporting the view that pharmacoperones will become powerful weapons in our therapeutic arsenal [21]. For this reason we have created a generalizable screening technique that allows identification of specific pharmacoperones from chemical libraries.

## Results

### Physiological Significance of the Targets Selected

The V2 receptor (V2R, also known as the arginine vasopressin receptor) is expressed in the distal convoluted tubule and the collecting ducts of the kidney. V2R responds to vasopressin by stimulating mechanisms that concentrate the urine and maintain water homeostasis in the organism. When the function of V2R is lost due to mutation, the disease nephrogenic diabetes insipidus (NDI) results. The gonadotropin-releasing hormone receptor (GnRHR, also known as the luteinizing hormone releasing hormone receptor) resides primarily in the gonadotrope cells of the pituitary and is responsible for producing responses to hypothalamic GnRH, such as the releasing of the gonadotropins, luteinizing hormone (LH) and follicle stimulating hormone (FSH). When the function of this receptor is lost due to mutation, the disease hypogonadotropic hypogonadism (HH) results.

### Advantages of the Approach Selected

Prior to establishing the cell-based assays described, we considered a number of alternative approaches. Because the assay is based on the redistribution of mutant GPCRs from the ER to the plasma membrane, we were unable to rely on membrane-type assays (e.g., GTPγS assays); intact and functional cells are required. Because well-characterized antiserum for GPCRs cannot be presumed to exist for every GPCR *a priori* (and, in fact, no well-characterized antiserum is available for the hGnRHR) we chose not to utilize ELISA or Western blot techniques. Fluorescently tagged (i.e. GFP) or HA-tagged mutants were not used, as this alters routing [19] and FLIPR™-type assays were not used because of their difficulty to configure, potential artifacts and the need for expensive equipment.

Among the advantages of the approach we selected are that stable cell lines produce a reproducible response upon receptor stimulation and give rise to a high signal window. In transiently transfected cells a high proportion of non-transfected cells may be present. Untransfected cells reduce the maximum signal since they do not contribute to production of stimulated endpoint. In addition, stable cells are convenient since they do not require separate transfection for each experiment.

A special feature of the cell lines developed is that the GPCRs or GPCR mutants are expressed under the control of the tetracycline-controlled transactivator (tTA). The tetracycline-regulated expression system is based on two components: a Tet-dependent transcription activator (tTA), which is a fusion between the Tet repressor of transposon TN10 and transcription factor binding domains of the herpes simplex protein VP16, and secondly, a tTA-responsive promoter, composed of seven Tet repressor binding sites (TetO7) immediately upstream of an RNA polymerase II transcriptional start site of the cytomegalovirus IE promoter (CMVm). When both elements are present in the cell, tTA binds to TetO7 and activates transcription at its neighboring initiation site. In the absence of tetracycline, the GPCR is expressed. In the presence of tetracycline, the GPCR is not measurably expressed. This model allows use of the GPCR to measure signal in the HTS (i.e. no tetracycline) and the identical background cell, lacking the expressed GPCR, to serve as a negative control (i.e. with tetracycline), thereby isolating false positives that may activate other cellular functions than the GPCR target.

We selected two model mutants of the human gonadotropin releasing hormone and vasopressin 2 receptors. These mutants are known to be misrouted, misfolded proteins [21], hGnRHR[E^90^K] [22] and hV2R[L^83^Q] [23] and are naturally occurring in patients with hypogonadotropic hypogonadism and nephrogenic diabetes insipidus, respectively. Stable HeLa cells were created expressing sequences for these proteins under control of a tetracycline-off promoter.

For both assays, we chose to use assays for effector coupling (IP or cAMP production) since, *in vivo*, effector activation would be the best measure of stimulation of the model systems under study. One could imagine receptor mutants that bound ligand, but failed to couple to effector. For such mutants, measuring receptor numbers (i.e. a radioligand assay) would provide a misleading measure of functional receptors. Moreover the need to develop a HTS is better served by using IP or cAMP as a screening assay, since it is quicker, does not require the use of ^125^Iodine around robotic equipment when HTS is performed.

### Primary Screen Model and Negative Control

The primary screen for pharmacoperones is rescue (gain) of function of stable HeLa cells expressing the indicated mutants (hGnRHR[E^90^K] or hV2R[L^83^Q]). A common confusion revolves around the use of an antagonist for receptor rescue which is then activated by an agonist. It is important to note that the pharmacoperone (a receptor antagonist) is not present at the time of agonist challenge. The general protocols for treatment with known pharmacoperones and determination of the resultant signal is described in Methods. Cell-containing wells were treated in replicates of 5–9 and then subjected to statistical evaluation. The format and protocol of the Assay Validation Guidelines by the Eli Lilly & NIH Chemical Center (http://www.ncgc.nih.gov/guidance/manual_toc.html), have been used in the present study.

### Statistical Evaluation of the GnRHR Mutant Assay

#### Linearity

**Figure 1:**
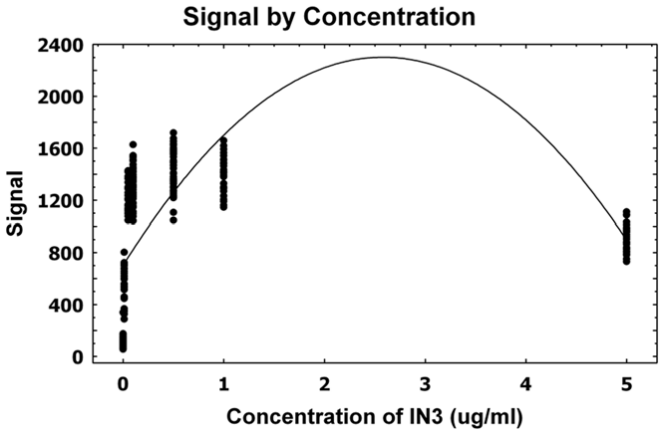
A scatterplot with regression fit for the GnRHR pharmacoperone assay. A linear regression analysis was attempted to evaluate the linearity/association of the dose-response of pharmacoperone and concentrations of dose by estimating a polynomial function.

Using known pharmacoperone drugs, the relation between the signal (“reads”) and dose concentrations was evaluated. Figure 1 suggests a quadratic relation between the reads and concentrations of pharmacoperone drug.

Based on this observation, a linear regression analysis was attempted to evaluate the linearity/association of the dose-response of pharmacoperone and concentrations of dose by estimating a polynomial function (Figure 1). The p-value for this model was <0.0001 as shown in Table 1, which indicates a significant relation between the dose-response and concentrations. The R-square value (0.37) suggests that only 37% the total variation is explained by the model. The estimated equation for this model is Signal = 701.94+1239.53×Concentration−240.18×Concentration^2^.

**Table 1:** Linear regression parameter estimates (GnRHR).

Variable	DF	Parameter Estimate	Standard Error	t-value	p-value
**Intercept**	1	701.94	33.16	21.17	<0.0001
**Concentration**	1	1239.53	95.48	12.98	<0.0001
**Concentration^2^**	1	−240.17	18.67	−12.86	<0.0001

**Figure 2:**
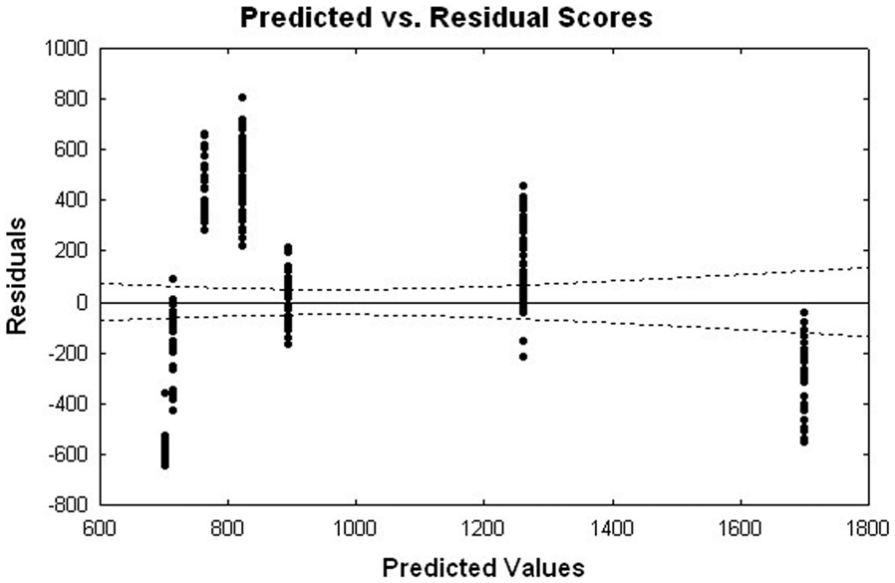
A residual plot for the GnRHR pharmacoperone assay. To assess the validity of the model assumptions, the plot of the Studentized residuals versus the predicted values, along with 95% confidence interval, is displayed.

**Figure 3:**
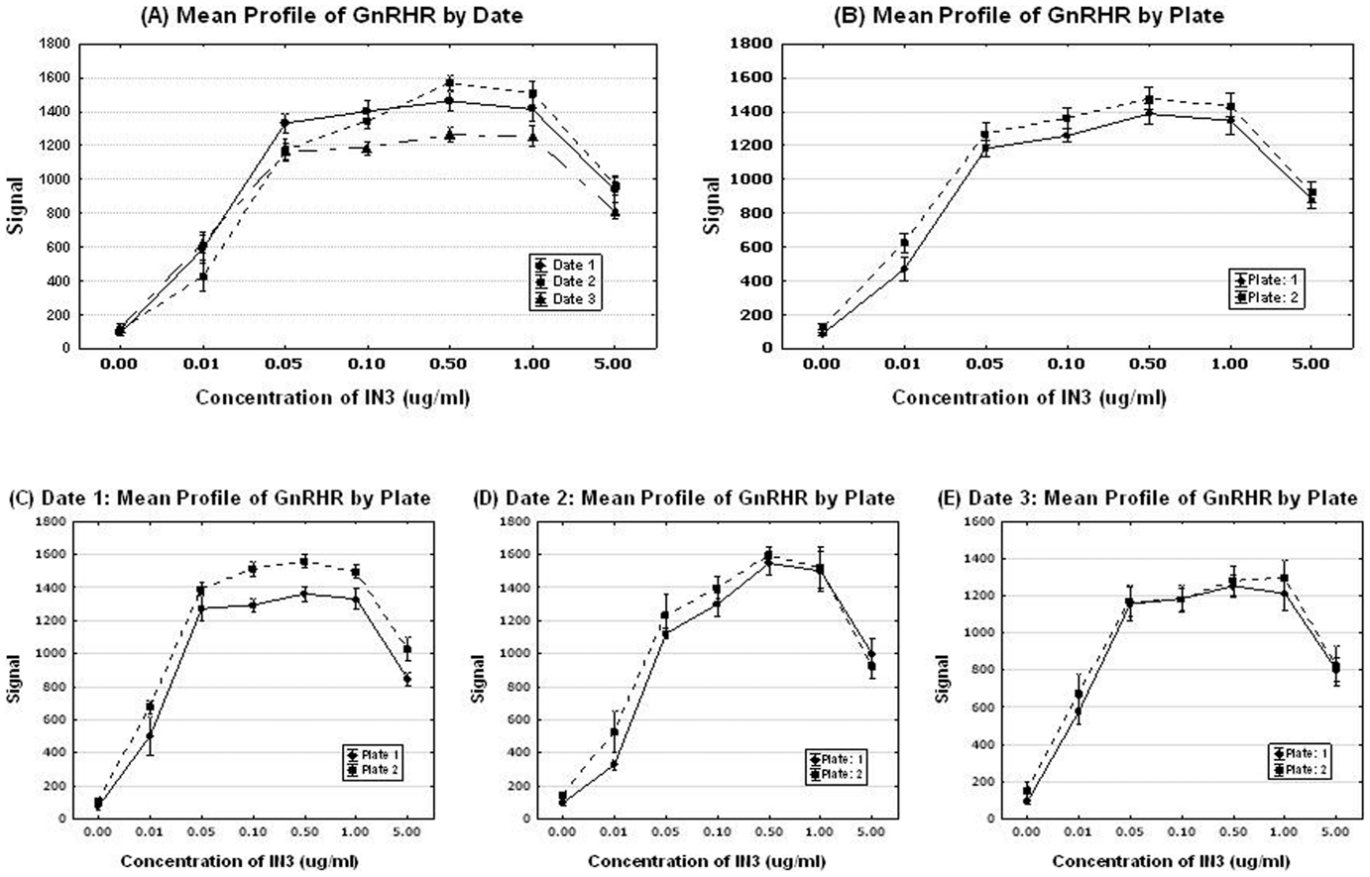
Mean profiles of signals by day and plate for the GnRHR pharmacoperone assay. Estimated equation for the model has a maximum gain of activity in stable HeLa cells at the 0.5 µg/ml dose by using the first order derivative.

The parameter estimates for both the linear and quadratic terms of concentrations were statistically significant with p-values<0.0001 (Table 1). To assess the validity of the model assumptions, the plot of the Studentized residuals versus the predicted values, along with 95% confidence interval, is performed and it is displayed in Figure 2. This figure exhibits systematic trends, suggesting that relation between the dose and read may be non-linear. We attempted some transformations and non-linear fits, but these alternative approaches have provided similar residual patterns. The estimated equation for the model has a maximum gain of activity in stable HeLa cells at the 0.5 µg/ml dose by using the first order derivative (Figure 3).

This estimated equation demonstrates that the signal will monotone increase as concentration increases until it reaches up to 0.5 µg/ml, while the signal will decrease as concentration increases for the concentration larger than 0.5 µg/ml.

#### Precision: Summary Signal & Plate Acceptance Criteria

Table 2 contains the descriptive statistics including averages (AVG), standard deviations (SD), standard errors (SE), and coefficient of variation (CV) for this assay. The CV was calculated taking into account the number of wells, N, 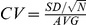. CV values for this study range from 2% to 11% which are smaller than the acceptance criterion (20%) by the assay validation guide line by the Eli Lilly & NIH Chemical Center (http://www.ncgc.nih.gov/guidance/manual_toc.html).

**Table 2:** Descriptive Statistics of Signals by Concentrations (GnRHR).

Concentration (µg/ml)	N	OFF				ON			
		Mean	Std Dev	Std Error	CV	Mean	Std Dev	Std Error	CV
**0.00**	60	46.55	9.19	1.19	0.03	110.00	43.22	5.58	0.05
**0.01**	30	44.69	7.28	1.33	0.03	547.73	136.68	24.95	0.05
**0.05**	30	47.18	7.87	1.44	0.03	1224.59	109.77	20.04	0.02
**0.10**	54	46.96	8.76	1.19	0.03	1311.93	139.09	18.93	0.01
**0.50**	54	48.28	6.87	0.94	0.02	1433.49	159.36	21.69	0.02
**1.00**	30	45.80	7.39	1.35	0.03	1393.15	138.98	25.37	0.02
**5.00**	30	35.41	6.29	1.15	0.03	903.96	105.08	19.18	0.02

#### Uniformity Assessment

Temporal uniformity and plate uniformity assessment. Repeated measures analysis of variance (ANOVA) was used to assess the effects of different dates of experiments and the marginal effects on plates in addition to concentrations. Line plots (Figures 3A–E) display the patterns of day to day variations as well as the plate to plate systematic variations. The figures suggest that there are some ‘date to date’ variations in the value on the Y-axis as well as ‘plate to plate’ variations, especially for higher concentrations (concentration >0.5 µg/ml).

There was a statistical significant effect of the dates (p-value = 0.04), and plates effect was not significant (p-value>0.05). These results are consistent with above tables and graphs.

#### Spatial Uniformity Assessment

**Figure 4:**
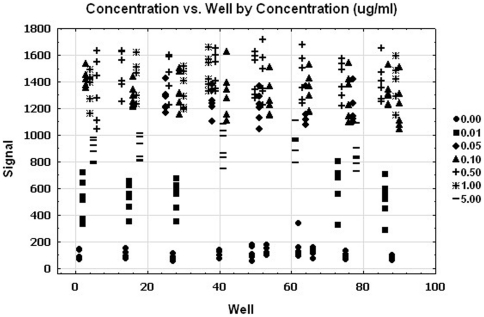
Spatial uniformity assessment of the GnRHR pharmacoperone assay. The signals are plotted against the well number. The wells are ordered by the rows and columns in order to explore any patterns of the edge, drift effect and other systematic source of variability.

The signals are plotted (Figure 4) against the well number. The wells are ordered by the rows and columns in order to explore any patterns of the edge, drift effect and other systematic source of variability. There was no significant edge effect (p-value = 0.79) using ANOVA. This figure suggests no trends (drift effect). The simple linear regressions were used to explore the trend in ‘signal over the location within the plate.’ There was no association between ‘the signal and location.’

### Statistical Evaluation of the V2R Mutant Assay

#### Linearity

**Figure 5:**
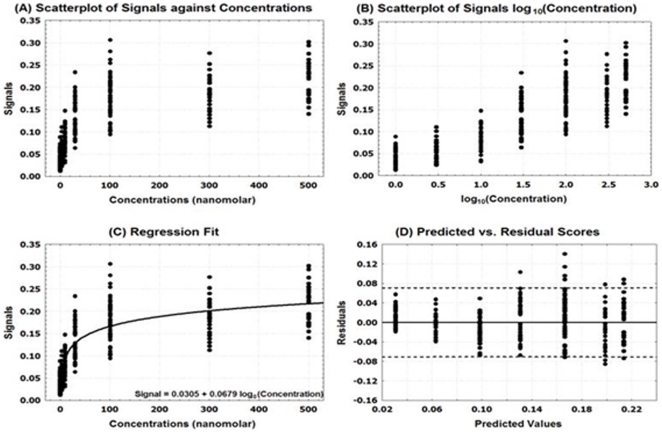
Linearity assessment for the V2R pharmacoperone assay. Using a pharmacoperone drug, the relation between the dose response and dose concentrations was evaluated.

Using pharmacoperone drugs, the relation between the dose response of pharmacoperone and dose concentrations of pharmacoperone drug V2R was evaluated. Figure 5A suggests that there is a near linear relation between the signals and concentrations with increased variation for higher concentrations.

Based on these observations, linear regression was attempted to evaluate the linearity/association of the dos-response of pharmacoperone by estimating a linear function. The linear regression models did not fit data very well. We attempted a log transformation with base 10 for the concentrations. Figure 5B displays a strong linear relation between the signal and the log transformed concentration.

Therefore, a linear regression was performed to evaluate the linearity/association of the dos-response of pharmacoperone by estimating a linear function of signals as a function of log_10_ (concentrations). Figure 5C displays the result of linear regression.

There was significant relation between the signals and the log transformed concentrations (p-value<0.0001). The R-square value indicates that the model accounts for 76% of the variation in the signals. Table 3 summarizes the parameter estimates along with their descriptive statistics. The null hypothesis that the slope is 0 was rejected at the level of 0.05 significance (p-value<0.0001). The fitted equation for this model is Signal = 0.0305+0.0679×log_10_(Concentration).

**Table 3:** Linear regression parameter estimates (V2R).

Variable	DF	Parameter Estimate	Standard Error	t-value	p-value
**Intercept**	1	0.0305	0.0037	8.29	<.0001
**Log_10_(Concentration)**	1	0.0679	0.0023	30.19	<.0001

The plot of the Studentized residuals versus the predicted values is displayed in Figure 5D. When a model provides a good fit and does not violate any model assumptions, this type of residual plot exhibits no marked pattern or trend. This residual plot exhibits no specific trends, indicating that model fits well.

#### Precision: Summary Signal & Plate Acceptance Criteria

Tables 4 shows the descriptive statistics including averages (AVG), standard deviations (SD), standard errors (SE), and coefficient of variation (CV). These were calculated as described above for the GnRHR mutant. CV values for this study range from 3.4% to 7.1% which are well below the acceptance criterion.

**Table 4:** Descriptive Statistics of Signals by Concentrations (V2R).

Concentration (nM)	N	Mean	Std Dev	Std Error	CV
0	60	0.04	0.02	0.00	0.07
3	30	0.06	0.02	0.00	0.07
10	30	0.09	0.03	0.01	0.06
30	54	0.13	0.04	0.01	0.04
100	54	0.18	0.05	0.01	0.03
300	30	0.19	0.04	0.01	0.04
500	30	0.21	0.04	0.01	0.04

#### Uniformity Assessment

**Figure 6:**
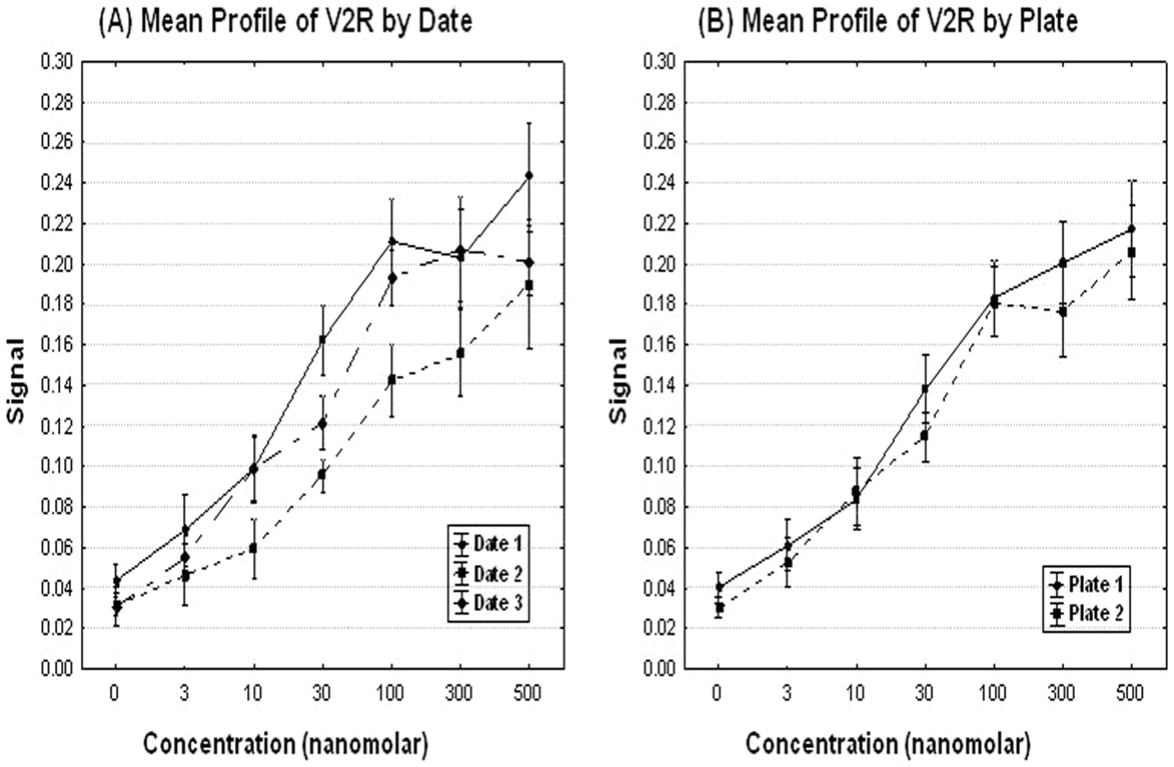
Mean profiles of signals by day and plate for the V2R pharmacoperone assay. Line plots were performed to reveal patterns of date by date variations, and plate to plate systematic variations.

Temporal uniformity and plate uniformity assessment. Line plots (Figures 6A and B) can reveal patterns of date by date variations, and plate to plate systematic variations.

There was a statistically significant effect of the date of experiment (p-value = <0.0001), and plate effect (p-value<0.001) using ANOVA. By inspection, this effect will not impair the ability of the assay to detect positives, but suggests that internal controls should be included for each date of experiment. It is also possible to compare data between dates if the discrimination level for differences is modestly increased.

#### Spatial Uniformity Assessment

**Figure 7:**
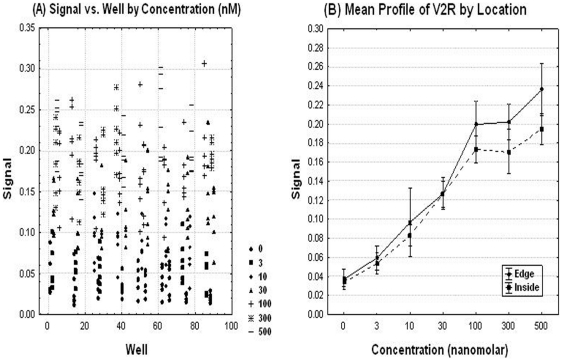
Spatial uniformity assessment for the V2R pharmacoperone assay. The signal was plotted against well number, where the wells are ordered by row first then by column in order to explore any pattern of edge, drift effect and other systematic source of variability.

The signal was plotted against well number, where the wells are ordered by row first then by column in order to explore any pattern of edge, drift effect and other systematic source of variability (Figures 7A and B). This figure suggests no trends (drift effect). The simple linear regressions were used to explore the trend in signals over the location (wells). There was no significant association between signals and location (p-value = 0.33).

### Assessment of Compounds that Would Not Be Expected to Provide a Signal in the Assays

**Figure 8:**
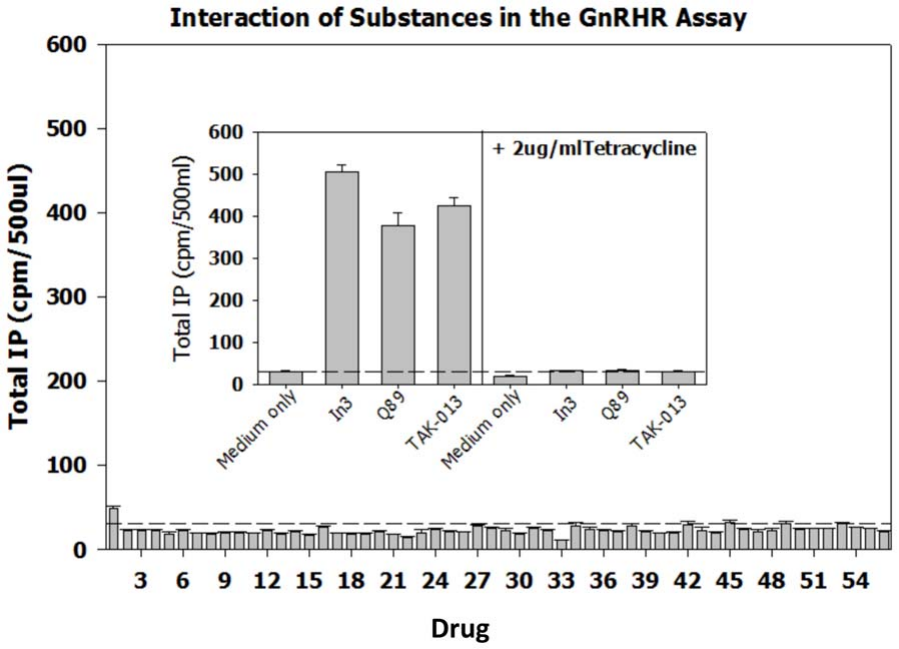
Performance of non-specific (expected negative controls) and positive controls for the GnRHR pharmacoperone assay. The non-specific compounds were selected from reagents that alter the level of cyclic nucleotides, block Ca2^+^ and Na channels, inhibit calmodulin, inhibit transcription, activate a range of receptors, cross link membrane proteins and otherwise perturb the cell. The dotted lines parallel to the X-axis show the response of the cells in the presence of medium only (i.e., no added drugs). The drugs, used at a concentration of 1 µg/ml were: 1. Urotensin II (neurosecretory peptide); 2. Octreotide Related Peptide; 3. Somatostatin; 4. Bombesin; 5. Calcitonin (salmon); 6. Growth Hormone Releasing Factor; 7. Thyrotropin Releasing Factor; 8. Galanin (human); 9. NPSF-Amide (SLAAPQRF-NH2); 10. Neuromedin U (rat); 11. BI 679 (the growth hormone releasing peptide, hexarelin); 12. Adiponectin (a hormone with broad impact on metabolism); 13. 1-(3-Dimethylaminopropyl)-3-Ethylcarbodiimide (a water-soluble protein crosslinker); 14. O2′-Monosuccinyl Guanosine 3′-5′-Cyclic Monophosphate Tyrosine Methyl Ester (cGMP analog); 15. 1-Ethyl-3-(3-Dimethylamino-Propyl)Carbodiimide-HCl (a water-soluble protein crosslinker); 16. D-β-3,4-dihydroxy-Phenylalanine (D-DOPA); 17. L-Noradrenaline; 18. Trifluoperazine (calmodulin antagonist); 19. Histone (from calf thymus) Type II-S (a basic protein); 20. N6-2′-O-Dibutyryladenosine 3′-5′-Cyclic Monophosphate (a cAMP analog); 21. Spermine; 22. Ouabain Octahydrate (Strophanthin-G) (sodium ion channel antagonist); 23. 3-Hydroxytyramine; 24. p-Nitrophenyl Phosphate; 25. 8-(4-Chlorophenylthio)-Adenosine 3′:5′-Cyclic Monophosphate; 26. Carbonyl Cyanide m-Chlorophenylhydrazone; 27. Guanosin-5′-triphosphate; 28. Adenylyl-imidodiphosphate (AMP-PNP); 29. p-Nitrophenyl-β-D-Galactopyranoside; 30. Cytochrome-C; 31. Concanavalin A (a plant lectin that interacts with plasma membrane glycoproteins); 32. L-1-Tosylamide-2-Phenyl-Ethylchloromethyl Ketone (inhibitor of trypsin-like enzymes); 33. Actinomycin D (transcription inhibitor); 34. β -Nicotinamide Adenine Dinucleotide (β-NAD); 35. Adenosine 5′-Monophosphoric Acid; 36. Nifedipine (Ca2+ ion channel antagonist); 37. D600 (Ca2+ ion channel antagonist); 38. 2-n-Propyl-Amino-indine; 39. Veratrine (Na channel inhibitor); 40. Vinblastine (microtubule inhibitor); 41. Cytochalasin D (blocks cellular actin polymerization); 42. Polyinosinic-Polycytidylic Acid (Poly[I]-Poly[C]) polymer; 43. Forskolin (activator or adenylate cyclase); 44. A23187 (Ca2+ ionophore); 45. SQ23,377 Ionomycin (Ca2+ ionophore); 46. U-73343 (inhibitor of inositol phosphate metabolism); 47. Creatine Kinase; 48. Deferoxamine Mesylate (metal chelator); 49. Flavin Mononucleotide; 50. [-]-Norepinephrine; 51. Adenosine-3,5′-Cyclic Monophosphothioate, Rp-Isomer; 52. N-nitro-L-arginine L-NAME Methyl Ester; 53. Acyline (GnRH peptide antagonist); 54. Asp2-GnRH (GnRH analog that binds GnRHR mutant E^90^K); 55. Buserelin (GnRHR peptide agonist); 56. Control (medium only); 57. In3 (non-specific for the V2R assay; non-peptide GnRH antagonist, structure [28]); 58. Q89 (non-specific for the V2R assay; non-peptide GnRH antagonist, structure [28]); 59. TAK-013 (non-specific for the V2R assay; non-peptide GnRH antagonist, structure [28]); 60. SR121463B (non-specific for the GnRHR assay, non-peptide V2R antagonist).

**Figure 9:**
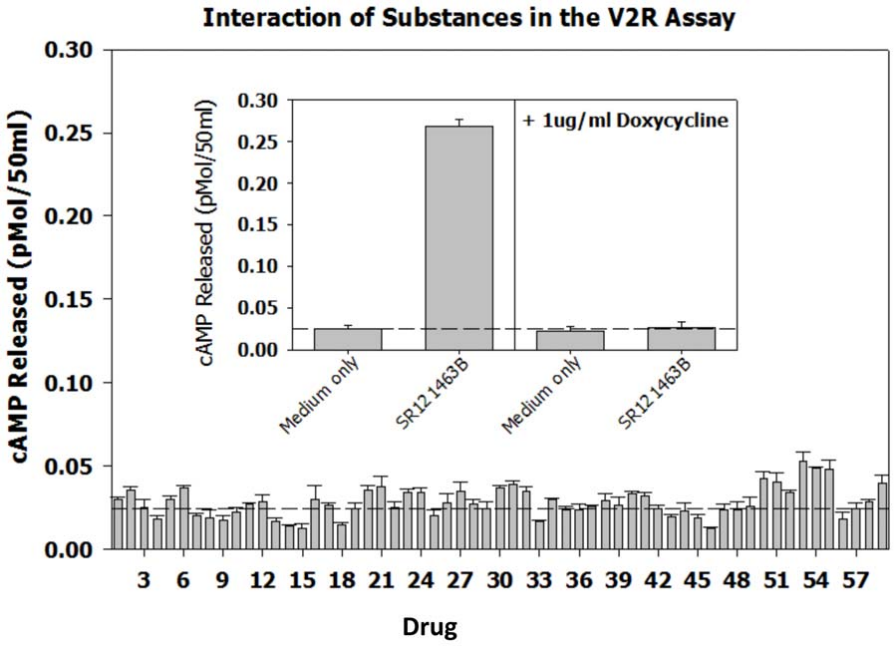
Performance of non-specific (expected negative controls) and positive controls for the V2R pharmacoperone assay. The non-specific compounds were selected from reagents that alter the level of cyclic nucleotides, block Ca2^+^ and Na channels, inhibit calmodulin, inhibit transcription, activate a range of receptors, cross link membrane proteins and otherwise perturb the cell. The dotted lines parallel to the X-axis show the response of the cells in the presence of medium only (i.e., no added drugs). The drugs, used at a concentration of 1 µg/ml were: 1. Urotensin II (neurosecretory peptide); 2. Octreotide Related Peptide; 3. Somatostatin; 4. Bombesin; 5. Calcitonin (salmon); 6. Growth Hormone Releasing Factor; 7. Thyrotropin Releasing Factor; 8. Galanin (human); 9. NPSF-Amide (SLAAPQRF-NH2); 10. Neuromedin U (rat); 11. BI 679 (the growth hormone releasing peptide, hexarelin); 12. Adiponectin (a hormone with broad impact on metabolism); 13. 1-(3-Dimethylaminopropyl)-3-Ethylcarbodiimide (a water-soluble protein crosslinker); 14. O2′-Monosuccinyl Guanosine 3′-5′-Cyclic Monophosphate Tyrosine Methyl Ester (cGMP analog); 15. 1-Ethyl-3-(3-Dimethylamino-Propyl)Carbodiimide-HCl (a water-soluble protein crosslinker); 16. D-β-3,4-dihydroxy-Phenylalanine (D-DOPA); 17. L-Noradrenaline; 18. Trifluoperazine (calmodulin antagonist); 19. Histone (from calf thymus) Type II-S (a basic protein); 20. N6-2′-O-Dibutyryladenosine 3′-5′-Cyclic Monophosphate (a cAMP analog); 21. Spermine; 22. Ouabain Octahydrate (Strophanthin-G) (sodium ion channel antagonist); 23. 3-Hydroxytyramine; 24. p-Nitrophenyl Phosphate; 25. 8-(4-Chlorophenylthio)-Adenosine 3′:5′-Cyclic Monophosphate; 26. Carbonyl Cyanide m-Chlorophenylhydrazone; 27. Guanosin-5′-triphosphate; 28. Adenylyl-imidodiphosphate (AMP-PNP); 29. p-Nitrophenyl-β-D-Galactopyranoside; 30. Cytochrome-C; 31. Concanavalin A (a plant lectin that interacts with plasma membrane glycoproteins); 32. L-1-Tosylamide-2-Phenyl-Ethylchloromethyl Ketone (inhibitor of trypsin-like enzymes); 33. Actinomycin D (transcription inhibitor); 34. β -Nicotinamide Adenine Dinucleotide (β-NAD); 35. Adenosine 5′-Monophosphoric Acid; 36. Nifedipine (Ca2+ ion channel antagonist); 37. D600 (Ca2+ ion channel antagonist); 38. 2-n-Propyl-Amino-indine; 39. Veratrine (Na channel inhibitor); 40. Vinblastine (microtubule inhibitor); 41. Cytochalasin D (blocks cellular actin polymerization); 42. Polyinosinic-Polycytidylic Acid (Poly[I]-Poly[C]) polymer; 43. Forskolin (activator or adenylate cyclase); 44. A23187 (Ca2+ ionophore); 45. SQ23,377 Ionomycin (Ca2+ ionophore); 46. U-73343 (inhibitor of inositol phosphate metabolism); 47. Creatine Kinase; 48. Deferoxamine Mesylate (metal chelator); 49. Flavin Mononucleotide; 50. [-]-Norepinephrine; 51. Adenosine-3,5′-Cyclic Monophosphothioate, Rp-Isomer; 52. N-nitro-L-arginine L-NAME Methyl Ester; 53. Acyline (GnRH peptide antagonist); 54. Asp2-GnRH (GnRH analog that binds GnRHR mutant E^90^K); 55. Buserelin (GnRHR peptide agonist); 56. Control (medium only); 57. In3 (non-specific for the V2R assay; non-peptide GnRH antagonist, structure [28]); 58. Q89 (non-specific for the V2R assay; non-peptide GnRH antagonist, structure [28]); 59. TAK-013 (non-specific for the V2R assay; non-peptide GnRH antagonist, structure [28]); 60. SR121463B (non-specific for the GnRHR assay, non-peptide V2R antagonist).

As a test of the specificity of the two assays and to compare the signal to noise ratio for known positives with non-specific compounds, a broad range of substances were evaluated in the assays (Figures 8, the GnRHR pharmacoperone assay and Figure 9, the V2R pharmacoperone assay). The non-specific compounds were selected from reagents that alter the level of cyclic nucleotides, block Ca^2+^ and Na channels, inhibit calmodulin, inhibit transcription, activate a range of receptors, cross link membrane proteins and otherwise perturb the cell. In both assays, no non-specific compound produced more than 2× signal above background, which the positive control signals were on the order of 10× above basal. This indicates that these assays produce a satisfactory range of discrimination that enables the determination of “positives.”

## Discussion

Pharmacoperones are small molecules that enter cells and serve as a “molecular scaffold” to promote correct folding of otherwise-misfolded mutant proteins [22], [24]. Because these drugs are frequently selected from candidates that were originally identified as target specific antagonists, these also show high target specificity as pharmacoperones, although competition for endogenous ligands is a therapeutic complication. Accordingly we sought to develop assays that would identify molecules that were not necessarily agonists or antagonists.

Although the use of pharmacoperone drugs *in vivo* is very recent and has not had the benefit of dose regime optimization, there are some *in vivo* successes that suggest the value of this approach. In a mouse model, Pey et al. [25] used compounds obtained from a chemical screen to treat rodents with phenylketonuria (PKU), an inherited metabolic disease caused by mutations in phenylalanine hydroxylase (PAH). This enzyme converts Phe to Tyr. Presently, restriction of access to Phe is an accepted therapy in humans. When WT-PAH or PKU-associated mutants were transiently expressed, treatment with such compounds increased PAH activity by up to 100%. This effect was associated with an increase in PAH synthesis and a decrease in its degradation. These compounds were effective when given orally and were able to stabilize PAH in the liver, increasing PAH activity and protein levels.

Another success involved patients with X-linked nephrogenic diabetes insipidus. Mutant vasopressin 2 receptors in NDI result in misrouted proteins that are trapped in the ER, degraded and do not reach the plasma membrane in the collecting ducts of the kidney where they would normally reabsorb water. *In vitro* studies indicated that a non-peptide V1a receptor antagonist rescued cell surface expression and function of mutant V2 receptors. When applied *in vivo*, a short-term treatment with a V1a receptor antagonist showed that patients given this molecule decreased both 24-h urine volume and water intake. Maximum increase in urine osmolality was observed on day 3 and sodium, potassium, and creatinine excretions and plasma sodium were constant throughout the study [26].

It is interesting and useful to note that, within a family of mutants of a single receptor, pharmacoperone drugs that rescue one mutant, often rescue most other mutants, even if the mutations are located distally from one another [3], [23]. This observation suggests that pharmacoperones bind at multiple sites and stabilize a conformation that is acceptable to the cell's quality control system, allowing them to traffic to the plasma membrane. Moreover, pharmacoperones from different chemical classes appear to bind to many of the same sites [27], [28]. These observations suggest that pharmacoperone drugs identified in the screens described here will identify lead structures that will be useful in treatment of human and animal mutational disease.

These studies describe and characterize a primary screen, relying on two model systems, by which chemical libraries can be examined for pharmacoperone drugs. The application of the tetracycline-controlled transactivator enables the identical line to be used as a negative screen, excluding drug candidates that show a response as a result of acting through a non-specific target. Because these targets are causally and mechanistically associated with pathophysiological responses, pharmacoperone drugs are likely to result in valid therapeutic approaches. The present study supports the statistical validity of the HTS screen for these compounds in two different model systems.

## Materials and Methods

### Stable (tTA+GPCR) Cells

The stable HeLa (tTA; tetracycline-controlled transactivator) cell line was obtained from Peter Seeburg (Max-Planck-Institut für Medizinische Forschung, Molekulare Neurobiologie Jahnstraße 29, 69120 Heidelberg, Germany). Cells were maintained in DFG growth medium (DMEM/10%FCS/20 µg/ml Gentamicin) and grown at 37°C, 5% CO_2_ in a humidified atmosphere until about 90% confluent. The cells were washed with Dulbecco's PBS, and then trypsinized to detach the cells. Growth medium will be added to the cells to dilute out the trypsin which will be centrifuged to pellet the cells. The hGnRHR[E^90^K] or hV2R[L^83^Q] mutants (in pTRE2-Hygromycin vector) were transfected into the stable HeLa (tTA) cell line. Selection antibiotics were used at 400 µg/ml G418 plus 200 µg/ml Hygromycin. Single colonies were selected and screened for expression of the mutant GPCRs. The dual stable cell lines were maintained using 200 µg/ml G418 plus 100 µg/ml Hygromycin in growth medium. Sub-cloning was used to select the best-expressing lines.

### Selection of Endpoint Measures

For both assays, we chose to use assays for effector coupling, (IP or cAMP production) since, *in vivo*, effector activation would be the best measure of stimulation (i.e. biological responses) of the model systems under study. One could imagine receptor mutants that bound ligand, but failed to couple to effector, for example. For such mutants, measuring receptor numbers (i.e. a radioligand assay) would provide a misleading measure of functional receptors.

### V2R Mutants: Stably Transfected HeLa Cells for measuring cAMP (tTA+hL^83^Q-V2R)

10^4^ cells per well were plated in 125 µl with or without 1 µg/ml Doxycycline in a 96-well plate. Fresh Doxycycline was added at least every 48 h to suppress the expression of the vector. Approximately 54 h after plating the cells, the cells were treated with DMSO (vehicle) or the rescue drug (SR121463B, Sanofi-Synthélabo Recherche) at 3×10^−9^, 1×10^−8^, 3×10^−8^, 1×10^−7^, 3×10^−7^, and 5×10^−7^ M prepared in 1% DMSO (final) and incubated for 16 h at 37 C. The cells were washed 3 times with 150 µl DBG containing 1% DMSO. The first 2 washes were 10 min at 37 C, then the last wash was a 20 min wash at 37 C. The cells were stimulated with 10^−6^ M vasopressin (Bachem) or media alone for 30 min. The media was removed and added to a tube containing 12.5 µl of 10 mM Theophylline and were boiled for 10 min.

### GnRHR Mutants: Stably Transfected HeLa Cells for measuring Inositol Phophates (tTA+hE^90^K-GnRHR)

10^4^ cells per well were plated in 125 µl with or without 2 µg/ml Tetracycline. After 24 h, the medium was changed and fresh Tetracycline was added for another 24 h period. The cells were then treated with DMSO (vehicle), 0.01, 0.05, 0.1, 0.5, 1, or 5 µg/ml In3 (Merck) prepared in 1% DMSO (final) and incubated for 4 h at 37 C with or without 2 µg/ml Tetracycline. The cells were then washed twice with 175 µl of DBG containing 1% DMSO with or without 2 µg/ml Tetracycline using a 10 min incubation at room temp for each wash. The cells were pre-loaded with 125 µl ^3^H-inositol (4 µCi/ml) with or without 2 µg/ml Tetracycline for 18 h in inositol free DMEM. The cells were washed with 175 µl of inositol free DMEM/5 mM LiCl with or without 2 µg/ml Tetracycline and stimulated with 10^−7^ M Buserelin with or without 2 µg/ml Tetracycline for 2 h. Total IPs were determined as previously described [29].

### Known Pharmacoperones

Known receptor-specific pharmacoperones were selected based on previous demonstration of efficacy. IN3 ((2*S*)-2-[5-[2-(2-azabicyclo[2.2.2]oct-2-yl)-1,1-dimethyl-2-oxo-ethyl]-2-(3,5-dimethylphenyl)-1*H*-indol-3-yl]-*N*-(2-pyridin-4-ylethyl)propan-1-amine) was used for the GnRHR mutant [22] and SR121463B (1-[4-(N-tert-butylcarbamoyl)-2-methoxybenzenesulfonyl]-5-ethoxy-3-spiro-[4-(2-morpholinoethoxy)cyclohexane]indol-2-one, fumarate) was used for the V2R mutant [23]. These were gifts of Merck and Company and Sanofi Recherche, Exploratory Research Department, Toulouse, France, respectively. Each is highly selective for the corresponding receptor and each binds in the nM range.

### Negative Screens

The negative screen is important for recognizing false positives. We have chosen to create stable cell lines in which the tetracycline-controlled transactivator controls expression of the mutant GPCRs. This transactivator shuts the gene off in the presence of this antibiotic [30]. We observed that there is literally no measurable expression, as assessed by protein expression or realtime PCR (unpublished), in the presence of tetracycline because the mutant GPCRs are under the control of this transactivator. Accordingly, cells cultured in the presence of tetracycline are substantially identical to the primary screen, *but lack expression of the target gene and gene product.* These cells (in the presence of tetracycline) will serve as an excellent negative control line. In both cases, coupling to second messenger is the measured endpoint. In all cases when cells were treated with tetracycline (or its analog doxycycline), the measured IP or cyclic AMP production was less than 2-fold basal and, generally, indistinguishable from basal; accordingly these data are not shown.

### Non-Specific Drugs, Peptides and other Chemicals Used

The following drugs were used (1 µg/ml) with non-specific actions:

1. Urotensin II (neurosecretory peptide); 2. Octreotide Related Peptide; 3. Somatostatin; 4. Bombesin; 5. Calcitonin (salmon); 6. Growth Hormone Releasing Factor; 7. Thyrotropin Releasing Factor; 8. Galanin (human); 9. NPSF-Amide (SLAAPQRF-NH2); 10. Neuromedin U (rat); 11. BI 679 (the growth hormone releasing peptide, hexarelin); 12. Adiponectin (a hormone with broad impact on metabolism); 13. 1-(3-Dimethylaminopropyl)-3-Ethylcarbodiimide (a water-soluble protein crosslinker); 14. O2′-Monosuccinyl Guanosine 3′-5′-Cyclic Monophosphate Tyrosine Methyl Ester (cGMP analog); 15. 1-Ethyl-3-(3-Dimethylamino-Propyl)Carbodiimide-HCl (a water-soluble protein crosslinker); 16. D-β-3,4-dihydroxy-Phenylalanine (D-DOPA); 17. L-Noradrenaline; 18. Trifluoperazine (calmodulin antagonist); 19. Histone (from calf thymus) Type II-S (a basic protein); 20. N6-2′-O-Dibutyryladenosine 3′-5′-Cyclic Monophosphate (a cAMP analog); 21. Spermine; 22. Ouabain Octahydrate (Strophanthin-G) (sodium ion channel antagonist); 23. 3-Hydroxytyramine; 24. p-Nitrophenyl Phosphate; 25. 8-(4-Chlorophenylthio)-Adenosine 3′:5′-Cyclic Monophosphate; 26. Carbonyl Cyanide m-Chlorophenylhydrazone; 27. Guanosin-5′-triphosphate; 28. Adenylyl-imidodiphosphate (AMP-PNP); 29. p-Nitrophenyl-β-D-Galactopyranoside; 30. Cytochrome-C; 31. Concanavalin A (a plant lectin that interacts with plasma membrane glycoproteins); 32. L-1-Tosylamide-2-Phenyl-Ethylchloromethyl Ketone (inhibitor of trypsin-like enzymes); 33. Actinomycin D (transcription inhibitor); 34. β -Nicotinamide Adenine Dinucleotide (β-NAD); 35. Adenosine 5′-Monophosphoric Acid; 36. Nifedipine (Ca^2+^ ion channel antagonist); 37. D600 (Ca^2+^ ion channel antagonist); 38. 2-n-Propyl-Amino-indine; 39. Veratrine (Na channel inhibitor); 40. Vinblastine (microtubule inhibitor); 41. Cytochalasin D (blocks cellular actin polymerization); 42. Polyinosinic-Polycytidylic Acid (Poly[I]-Poly[C]) polymer; 43. Forskolin (activator or adenylate cyclase); 44. A23187 (Ca^2+^ ionophore); 45. SQ23,377 Ionomycin (Ca^2+^ ionophore); 46. U-73343 (inhibitor of inositol phosphate metabolism); 47. Creatine Kinase; 48. Deferoxamine Mesylate (metal chelator); 49. Flavin Mononucleotide; 50. [-]-Norepinephrine); 51. Adenosine-3,5′-Cyclic Monophosphothioate, Rp-Isomer; 52. N-nitro-L-arginine L-NAME Methyl Ester; 53. Acyline (GnRH peptide antagonist); 54. Asp^2^-GnRH (GnRH analog that binds GnRHR mutant E^90^K); 55. Buserelin (GnRHR peptide agonist); 56. Control (medium only); 57. In3 (non-specific for the V2R assay; non-peptide GnRH antagonist, structure [28]); 58. Q89 (non-specific for the V2R assay; non-peptide GnRH antagonist, structure [28]); 59. TAK-013 (non-specific for the V2R assay; non-peptide GnRH antagonist, structure [28]); 60. SR121463B (non-specific for the GnRHR assay, non-peptide V2R antagonist).
